# A Chemometrics Approach Comparing Volatile Changes during the Shelf Life of Apple Juice Processed by Pulsed Electric Fields, High Pressure and Thermal Pasteurization

**DOI:** 10.3390/foods7100169

**Published:** 2018-10-17

**Authors:** Biniam Kebede, Pui Yee Lee, Sze Ying Leong, Vidya Kethireddy, Qianli Ma, Kemal Aganovic, Graham T. Eyres, Nazimah Hamid, Indrawati Oey

**Affiliations:** 1 Department of Food Science, University of Otago, P.O. Box 56, Dunedin 9054, New Zealand; biniam.kebede@otago.ac.nz (B.K.); carie1989@gmail.com (P.Y.L.); sze.leong@otago.ac.nz (S.Y.L.); vkethireddy@gmail.com (V.K.); graham.eyres@otago.ac.nz (G.T.E.); 2 Riddet Institute, Private Bag 11 222, Palmerston North 4442, New Zealand; 3 Faculty of Health and Environmental Science, Auckland University of Technology, Private Bag 9006, Auckland 1142, New Zealand; roy.ma@otago.ac.nz (Q.M.); nazimah.hamid@aut.ac.nz (N.H.); 4 German Institute of Food Technologies, P.O. Box 1165, D-49610 Quakenbrück, Germany; K.Aganovic@dil-ev.de

**Keywords:** high-pressure processing, pulsed electric fields, apple juice, shelf life, volatile, chemometrics

## Abstract

High-Pressure Processing (HPP) and Pulsed Electric Fields (PEF) processing technologies are being used increasingly on a commercial basis, with high-quality labelled fruit juices being one of the most important promotion strategies. Quality-related enzymes, which might still be active after HPP and PEF pasteurization, can cause undesirable aroma changes during storage. This study investigated volatile changes during the shelf life of PEF (15.5 kV/cm and specific energy of 158 kJ/L), HPP (600 MPa for 3 min), and thermally (72 °C for 15 s) pasteurized Jazz apple juices—up to five weeks. To have an increased insight into the volatile changes, an integrated instrumental (GC-MS) and data analysis (chemometrics) approach was implemented. Immediately after pasteurization, PEF processing resulted a better retention of odor-active volatiles, such as (*E*)-2-hexenal and hexyl acetate, whereas thermal processing lowered their amount. During refrigerated storage, these volatiles have gradually decreased in all processed juices. By the end of storage, the amount of these aroma relevant volatiles appears to still be higher in PEF and HPP pasteurized juices compared to their conventional counterparts. This study demonstrated the potential of advanced chemometric approaches to obtain increased insight into complex shelf life changes.

## 1. Introduction

One of the main challenges in the fruit juice industry is to produce juices with a flavor close to that of freshly squeezed fruits and that remains consistent during storage. This has led to the development and introduction of emerging processing technologies such as High-Pressure Processing (HPP) and Pulsed Electric Fields (PEF) that have been attracting a lot of research interest [1,2,3].

HPP involves the application of hydrostatic pressure, while high-voltage pulses are used in PEF processing [4,5]. HPP- and PEF-treated foods are generally claimed to have superior sensorial and nutritional quality compared to their thermally treated counterparts [1,6,7,8,9]. Today, both technologies are being used increasingly on a commercial basis, with high-quality labelled fruit juices being one of the most important promotion strategies [3,10,11,12]. However, HPP- and PEF-processed juices might have a limited shelf life since quality-related enzymes (e.g., polyphenol oxidases and peroxidase) are still active after HPP and PEF pasteurization, and can cause undesirable aroma changes during storage [9,13].

Apple juice is one of the most popular juices due to its pleasant organoleptic qualities and incredible health benefits [14,15]. Considering the importance of aroma compounds for apple juice quality, it is crucial to investigate the change in volatile compounds during processing and storage. Previously, some studies investigated the most potent odorant(s) contributing to apple juice aroma through linking the sensory attribute to a single (group of) compound(s) [16]. For instance, some esters have been identified as main contributors to the overall apple juice aroma. It is known that perceived odor is not due to a single (group of) volatile compound(s) but rather a result of a large number of volatile compounds [17]. Advanced analytical and data analysis procedures offer an opportunity to overcome these limitations [9].

The present work aims at comparing the impact of thermal, HPP, and PEF pasteurization technologies on the volatiles of Jazz apple juice after processing and during refrigerated storage of up to five weeks. The study on volatiles change was conducted systematically by integrating the currently available instrumental (GC-MS) and the state-of-the-art data analysis (chemometrics) techniques. The GC-MS data were analyzed using multivariate data analysis (MVDA) techniques, namely partial least squares (PLS) regression. To compare the process impact immediately after processing, a partial least squares‒discriminant analysis (PLS-DA) model was used. For the PLS-DA model, different processing techniques were used as categorical Y-variables. To investigate the volatile changes during shelf life, a PLS regression model was constructed, using the storage time as a continuous Y-variable. Compounds that are differently affected by a certain processing technology or compounds changing the most during storage, which are discriminant markers, were selected using a variable identification (VID) procedure. These selected compounds were linked to apple juice aroma in order to draw a conclusion about the relevance and consequences of the detected differences [9,18,19,20,21,22].

## 2. Materials and Methods

### 2.1. Sample Preparation

In this work, New Zealand Jazz apples were used. The apples (harvested in 2014) were transported to the Department of Food Science, Dunedin, New Zealand and stored at 4 °C. The apples were washed with 100 ppm chlorinated water for 1 min (Hypostat 135, Wilsons Chemicals, Christchurch, New Zealand) and then rinsed with distilled water to minimize contamination. The whole apples were juiced using a Breville Juice Fountain (model JE90, Breville, Sydney, Australia), sieved (0.5 mm) to remove the pulp and transported to a storage tank until processing. The tank was maintained at 4 °C.

### 2.2. Sample Pasteurization

Jazz apple juice was pasteurized with thermal, HPP, and PEF treatments, aiming to achieve short-term storage under refrigerated conditions. The details of the applied processing conditions for thermal, HPP, and PEF pasteurization can be found in Lee et al. [23,24]. The literature search revealed that there is still a lack of reliable microbial inactivation kinetic data during HPP and PEF processing. Therefore, the target microorganisms for HPP and PEF processing are not yet agreed upon. In this work, aiming for a fair comparison, the processing conditions for thermal, HPP and PEF technologies were selected to achieve a 4-log reduction of total plate counts.

The conventional thermal pasteurization was performed using a continuous tubular heat exchanger (inner diameter of 10 mm, length of 200 cm). The apple juice was treated at 72 °C for 15 s. Following the treatment, the samples were cooled down to 13 °C and packed into pre-sterilized polyethylene Whirl-Pak plastic bags under hygienic conditions.

For the HPP, the apple juice was pasteurized using industrial-scale HPP equipment (HPP 055, Multivac, Sepp Haggenmüller GmbH & Co., Wolfertschwenden, Germany). Prior to the treatment, the apple juice was vacuum-packed in pre-sterilized polyethylene Whirl-Pak plastic bags. For this equipment, water was used as a pressure medium. The inlet temperature of the water was maintained at 7–8 °C. During processing, the pressurization rate to reach 600 MPa was 125 MPa/min. The packed juice was then held at 600 MPa for 3 min. After the holding time, the pressure was released in a step-wise fashion to avoid leakage of the vacuum sealing.

For PEF treatment, the juice was first preheated to 30 °C using a continuous tubular heat exchanger. After preheating, the juice was subjected to PEF treatment (ELCRACK^®^, HVP-5, DIL, German Institute of Food Technologies, Quakenbrück, Germany). The processing conditions were: a pulse width of 20 µs, frequency of 48 Hz, flow rate of 16 L/h, an electric field strength of 15.5 kV/cm and a specific energy of 158 kJ/L. A continuous mode was applied using a co-linear treatment chamber, with an internal diameter of 10 mm and a gap of 10 mm between the electrodes (titanium, grade: 3.7035), using bipolar square wave pulses. Immediately after pasteurization, the juice was circulated in a chilled water jacket to cool it down to 19 ± 1 °C. Finally, the juice was packed under hygienic conditions into pre-sterilized polyethylene Whirl-Pak plastic bags.

### 2.3. Storage

Samples from all treatments were then stored at 4 °C for up to five weeks. At fixed points of storage time, samples of each treatment were taken out. The samples were transferred to polyethylene terephthalate plastic bottles, frozen in liquid nitrogen and stored in a freezer at −40 °C until instrumental analysis. The pH of stored samples was measured during storage as an indicator for microbial activity; the processed samples seemed to be stable during short-term storage in refrigerated conditions.

### 2.4. Volatile Analysis

The analysis of the apple juice volatile fraction was performed based on the procedure described by Aguilar-Rosas et al. [12] with some modifications. The thawed apple juice (5 mL) was placed in 20-mL vials fitted with a magnetic crimp cap with silicon septum seal (GERSTEL, Linthicum, MD, USA). In this study, 1,2-dichlorobenzene was used as an internal standard. The volatile analysis was conducted on a GC system (Trace GC Ultra, Thermo Scientific, Waltham, MA, USA) equipped with a Dual-Stage Quadrupole (DSQ) single mass spectrometer (Thermo Scientific). This analysis includes different steps: sample incubation, extraction and separation and detection. Sample incubation was carried out at 60 °C for 30 min under agitation at 250 rpm. Next, the headspace compounds were extracted using a solid phase microextraction (SPME) fiber coated with 30/50 µm divinylbenzene/carboxen/poly(dimethylsiloxane) (DVB/CAR/PDMS) (Supelco, Bellefonte, PA, USA) during 5 min. The extracted volatiles were then injected, in a split-less mode (1/5), into a GC injection port, which was set at an inlet temperature 200 °C. Chromatographic separation was carried with a VF-5ms low bleed/MS fused-silica capillary column (5%-phenyl-95%-dimethylpolysiloxane phase, 30 m × 0.32 mm × 0.50 μm) (Agilent Technologies, Santa Clara, CA, USA). Helium gas was used as carrier gas with a constant flow rate of 1.5 mL/min. The GC oven temperature program was as follows: the oven was initially held for 3 min at 35 °C, then raised to 170 °C at a rate of 5 °C/min and held for 2 min, then finally ramped to 250 °C and held for 3 min at this temperature. The mass spectra were obtained by electron ionization (EI) mode at 70 eV with a scanning range of *m/z* 30–400 and at a rate of 0.82 scan/s. MS ion source and quadrupole temperatures were 200 °C and 150 °C, respectively.

### 2.5. Data Analysis: Multivariate Data Analysis

The procedure established by Vervoort et al. [19] and Kebede et al. [21] was followed. The MVDA was carried out using Solo (Version 6.5, 2011, Eigenvector Research, Wenatchee, WA, USA). Prior to MVDA, the chromatograms were pre-processed using autoscaling. Autoscaling includes mean centering followed by standardization. For the latter, the variables were weighed by their standard deviation to give them equal variance. In this work, the process impact comparison was performed (i) immediately after processing and (ii) during storage. To compare the process impact immediately after processing, a PLS-DA model was constructed. For PLS-DA, the volatile compounds were considered as X-variables and the processing technologies were considered as categorical Y-variables. To study the evolution of the volatile fractions as function of storage time, a PLS regression model was built for each pasteurization technology. The shelf life changes were studied only for the processed samples, as the unprocessed/control samples were microbiologically unstable after one week of refrigerated storage. For PLS regression, the volatiles and storage time were considered as X- and Y-variables, respectively. For each model, the optimum number of latent variable (LV) that explain the maximum variance in the data with the minimum noise was selected. Based on the PLS models, bi-plots were generated to compare the differently processed or stored samples. Bi-plots provide a graphical representation of the similarities and/or differences between the samples. To identify volatile compounds responsible for these trends as a function of processing or storage, variable identification (VID) coefficients were calculated. With the VID procedure, each volatile compound was assigned with a coefficient between −1 and +1 per each processing or storage condition. To determine the most important ones, variables with an absolute VID value higher than 0.800 were selected. These discriminant compounds were then identified using NIST spectral library (NIST14, National Institute of Standards and Technology, Gaithersburg, MD, USA) [19,21].

## 3. Results and Discussion

The effect of thermal, HPPm and PEF pasteurization techniques on apple juice volatile fraction was investigated. In the first section, the process impact immediately after processing will be discussed. Next, the impact of refrigerated storage will be discussed.

### 3.1. Process Impact Comparison Immediately after Processing

**Figure 1:**
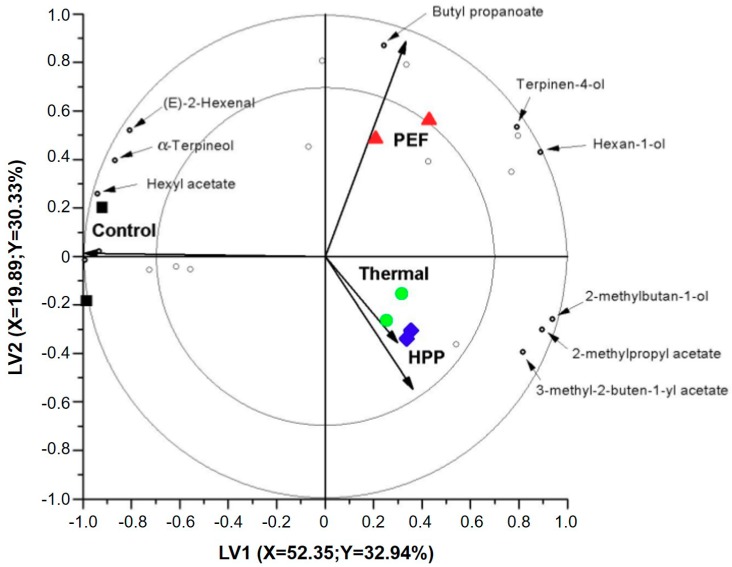
A PLS-DA biplot showing the comparison of volatile fraction of control/unprocessed apple juice (■), thermal (72 °C for 15 s) (●), HPP (600 MPa for 3 min) (♦) and PEF (15.5 kV/cm and specific energy of 158 kJ/L) (▲) pasteurized juices. The volatile compounds are represented with the open circles. Volatiles with amounts clearly different between the different samples (discriminant markers) are named. PLS-DA: partial least squares-discriminant analysis; HPP: High-Pressure Processing; PEF: Pulsed Electric Fields; LV: latent variable.

In this work, 21 volatile compounds were detected, in the fresh and processed samples, with the headspace GC-MS approach. These compounds include ten esters (*n*-propyl acetate, isobutyl acetate, butyl acetate, 2-methylbutyl acetate, ethyl 2-methylbutanoate, butyl propanoate, pentyl acetate, 3-methyl-2-buten-1-yl acetate, 2-methylpropyl butanoate and hexyl acetate); seven alcohols and terpene alcohols (2-methyl-1-propanol, 1-butanol, 2-methyl-1-butanol, 1-hexanol, 1-octanol, terpinen-4-ol and α-terpineol); three aldehydes (hexanal, (*E*)-2-hexenal and octanal); and one ketone (6-methyl-5-hepten-2-one). A PLS-DA model was used to compare the volatile fraction of differently pasteurized apple juices immediately after processing. Figure 1 shows a bi-plot using LV1 and LV2. On the plot, the four samples (control, PEF, thermal and HPP) and 21 volatile compounds (small open circles) are shown. Based on the distance between the samples on the plot, the similarity and/or difference between the differently processed samples can be investigated. From Figure 1, the first clear trend is that all processed samples are projected to the right side of the plot and far away from the unprocessed/control samples. Hence, immediately after processing, there is a clear effect of the applied pasteurization technologies on Jazz apple juice volatile fraction. This difference between the processed and unprocessed samples is explained by the first LV. The second trend on the plot is the difference among the pasteurization technologies. The second LV explains the difference between PEF pasteurized samples, on the one side, and thermal and HPP-pasteurized samples on the other side. Immediately after processing, PEF processed juices seem to have a distinct headspace fraction, whereas thermal and HPP processed juices seem to have a comparable headspace fractions.

A bi-plot also displays the correlation between the samples (control and processed) and individual volatile compounds. On the plot, if a volatile compound is positioned close to a certain sample this shows that it is detected with a higher amount in that particular sample compared to the other samples in the model. Volatiles that are projected in the opposite direction to a certain sample are detected in lower amounts in that sample compared to the other samples. As can be seen from Figure 1, a number of volatile compounds are detected in higher amounts in the control samples compared to the pasteurized juices, whereas few other compounds are detected in higher amounts in the processed samples. Hence, bi-plots provide relevant graphical information about the relation between volatile compounds and applied processing technologies. However, bi-plots only provide a graphical information and it is not straightforward to rank compounds based on their concentration in one processing technique compared to another one. For that reason, VID coefficients were calculated. Per sample, each volatiles were given a VID coefficient between −1 and +1. A positive VID coefficient in a certain sample shows a higher amount in that particular sample compared to the other samples and vice versa. Since the aim was to determine volatiles clearly affected by the applied processing or storage time, only those with an absolute VID value higher than 0.800 (discriminant volatiles) were selected and identified (Table 1, Figure 1).

In the control samples, seven volatile compounds were selected with the VID procedure. On the one side, three of these compounds, (*E*)-2-hexenal, α-terpineol and hexyl acetate were selected with a positive VID value. This shows that these compounds are detected in higher amounts in the control samples or in another words they are detected in lower amounts in the processed samples and thus are possibly decreased due to the applied pasteurization techniques. In line with that statement, two of these compounds, (*E*)-2-hexenal and α-terpineol, are detected at significantly lower amounts in thermally processed apple juices compared to other samples. (*E*)-2-hexenal is one of the volatile compounds reported to have a significant aroma relevance in apple juices. Hence, it seems that conventional thermal processing exerts a negative impact on some aroma relevant apple juice volatile compounds. Yi et al. [9] also reported that the aroma of apple juice seem to be more affected due to thermal processing compared to HPP, in particular causing increased formation of compounds responsible for cooked notes. On the other side, four volatiles, 2-methylbutan-1-ol, 2-methylpropyl acetate, hexan-1-ol and 3-methyl-2-buten-1-yl acetate, were selected with a negative VID values showing that these compounds are detected with a lower abundance in the control samples compared to processed ones. In relation to that, some of these volatiles were significantly increased after PEF and HPP pasteurization. The amount of 2-methylpropyl acetate seems to be increased after HPP. Hexan-1-ol, terpinen-4-ol and butyl propanoate were detected with higher amounts after PEF processing.

In general, immediately after processing, thermal processing seems to reduce the amount of some odor-relevant volatiles compared to HPP and PEF pasteurization technologies. However, since the quality of processed juices further changes during shelf life, in the next section, the processing impact was investigated during refrigerated storage.

### 3.2. Effect of Refrigerated Storage

**Figure 2:**
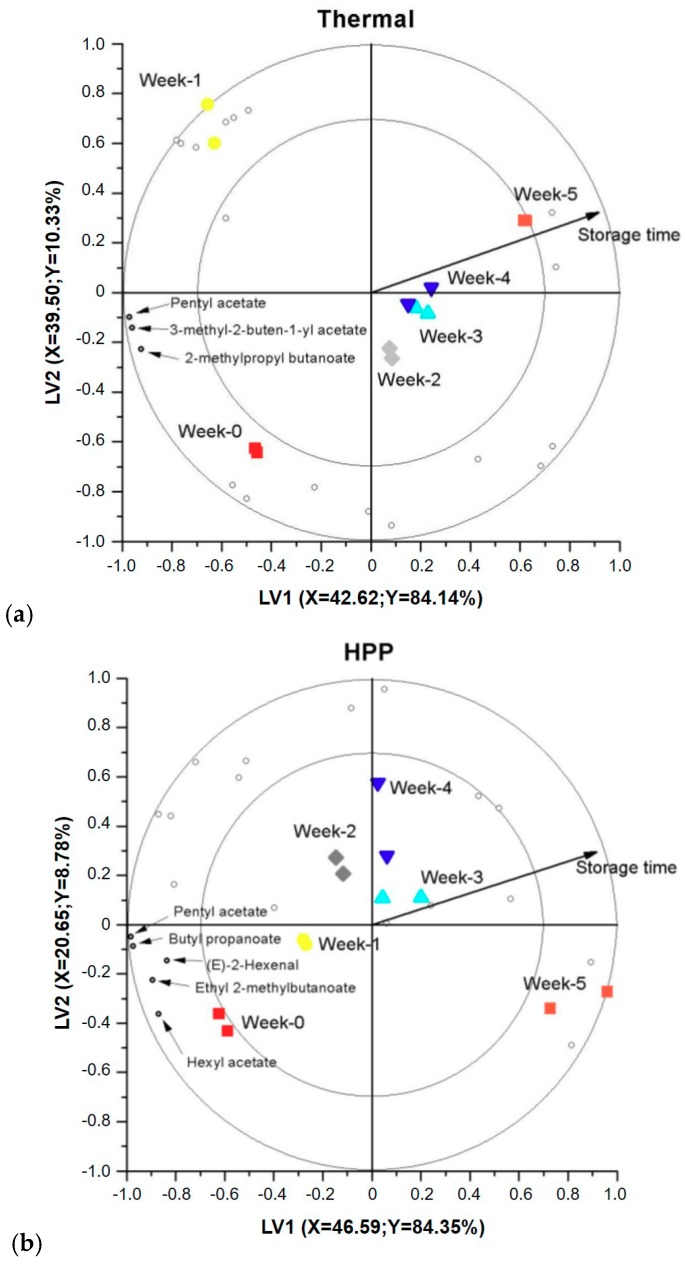

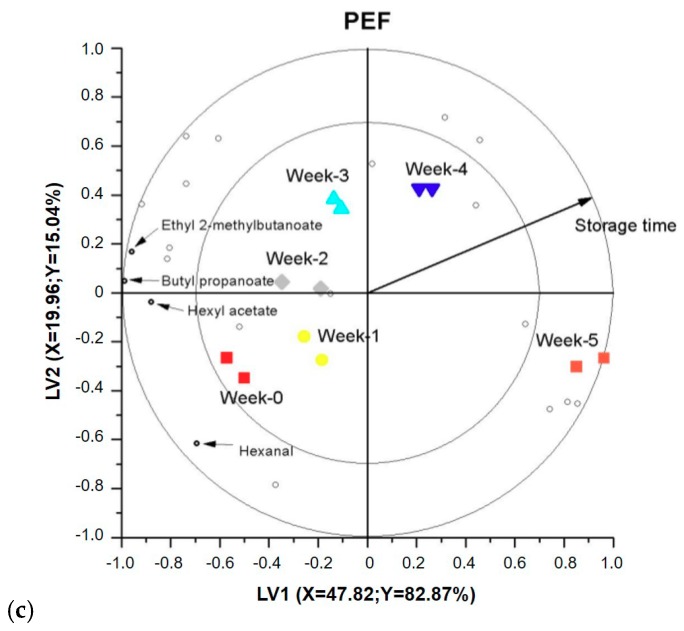
PLS-DA biplots showing the change in the volatile fraction of thermally (72 °C for 15 s) (**a**), HPP (600 MPa for 3 min) (**b**) and PEF (15.5 kV/cm and specific energy of 158 kJ/L) (**c**) pasteurized apple juice during refrigerated storage (from week 0 ■, 1 ●, 2 ♦, 3 ▲ to 4 ▼). The volatile compounds are represented with open circles. Volatiles clearly changing as a function of shelf life (which are discriminant markers) are named.

A PLS regression was used to evaluate the change in Jazz apple juice volatile fractions during refrigerated storage. For each processing condition, two latent variables adequately explained a considerable amount of the Y-variance (94%, 93% and 97% for thermal, HPP and PEF, respectively) (Figure 2a–c). Accordingly, for each processing conditions, a multivariate PLS regression model using two LVs was selected. The first obvious trend on all three bi-plots is that the apple juice volatile fraction clearly changes during refrigerated storage. This can be seen from the horizontal projection of apple juice volatile fractions from the left to the right side of the bi-plots. This dominant change during storage is adequately described by the first LV, as indicated in the respective axis (at least 80% Y-variance explained). Even though it is minimal, there is also a variation in the vertical direction in addition to the horizontal direction. This second variation on the plot is described by the second LV. On all three bi-plots, most of the volatiles are projected to the beginning of the shelf life, indicating that the amount of these compounds has decreased as a function of storage time. VID coefficients were calculated to determine volatiles significantly changed during storage. As can be seen from Table 2, three, five and four discriminant markers were selected in the thermal, HPP and PEF pasteurized juices, respectively.

The aroma of apple juice is mainly related to volatile compounds such as esters, alcohols, aldehydes, ketones and ethers [17]. Even though the literature search revealed more than 300 volatile compounds in apple juice, only 20–40 odor-active volatiles including ethyl-2-methyl butanoate, ethyl acetate, ethyl butanoate, (*E*)-2-hexenal and 1-butanol are considered as being responsible for apple juice aroma [16,17,25,26]. In the present work, the amount of some of these ester and aldehyde volatile compounds has significantly decreased during storage in all processed samples (see Table 2). This shows that the fresh, green and fruity note of apple juice seem to drop as a function of shelf life. Moreover, esters and aldehydes seem to be the main chemical groups significantly changing during shelf life in all thermal, HPP- and PEF-pasteurized apple juices (Table 2).

**Figure 3:**
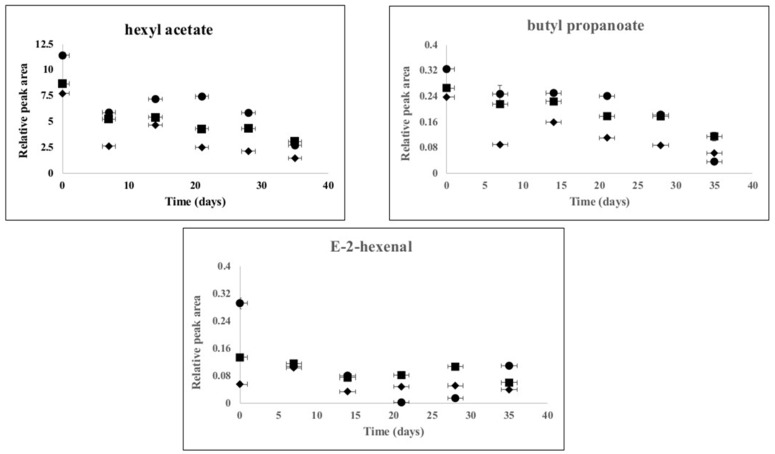
Change in the relative peak areas (peak area of compound/peak area of internal standard) of hexyl acetate, butyl propanoate and (*E*)-2-hexenal as a function of storage time at 4 °C in thermal (72 °C for 15 s) (♦), HPP (600 MPa for 3 min) (■) and PEF (15.5 kV/cm and specific energy of 158 kJ/L) (●) pasteurized apple juices. A standard deviation of two replication is included.

In the next step, to increase insight into the evolution of the changes of these compounds up on storage in the differently pasteurized apple juices, the GC-MS data was plotted as a function of time. As an example, the changes of hexyl acetate, butyl propanoate and (*E*)-2-hexenal are presented in Figure 3.

Immediately after processing, these volatile compounds were detected at higher levels in PEF and HPP pasteurized samples compared to thermally pasteurized samples. This observation is comparable with the discussion in the previous section that conventional thermal processing seems to have a negative impact on these aroma-relevant volatile compounds immediately after processing. During storage, these compounds have decreased in a similar fashion in all processed samples. Hence, the present work demonstrated that process impact comparison should be performed not only immediately after processing but also during storage. These observations are in line with research results previously reported by Vervoort et al. [19]. Moreover, it is noteworthy that the amount of these selected volatile compounds appears to be slightly higher in PEF and HPP pasteurized apple juices in comparison to their conventional counterparts by the end of storage (Figure 3). It is unsure whether the observed differences would be perceived by humans. However, since most of these volatiles are reported to be contributing to apple juice aroma, mild pasteurization by PEF or HPP could provide better aroma retention during storage.

## 4. Conclusions

This study demonstrated the potential of state-of-the-art chemometrics approach to have an increased insight into volatile changes during shelf life of PEF, HPP and thermal pasteurized apple juices. Immediately after processing, thermal processing lowered the amount of odor-active ester and aldehyde volatiles in comparison to PEF and HPP technologies. Consequently, at the end of storage, the amount of these aroma relevant volatiles appears to be still higher in PEF and HPP pasteurized juices compared to their conventional counterparts. Hence, mild pasteurization by PEF or HPP seems to provide a better retention of aroma-relevant volatiles during apple juice storage. Based on these results, it is difficult to evaluate how the observed modification of the volatile fraction will affect the overall apple juice aroma. Therefore, there is a need for a sensory analysis to understand how these changes will be appreciated.

## Figures and Tables

**Table 1:** Discriminant volatile markers in Jazz apple juice for control, thermal, HPP and PEF processing, which are selected by the VID procedure.

Processing	VID	Identity	RI
Control/Unprocessed	−0.932	2-methylbutan-1-ol	732
	−0.886	2-methylpropyl acetate	965
	−0.882	hexan-1-ol	869
	−0.809	3-methyl-2-buten-1-yl acetate	919
	0.820	(*E*)-2-hexenal	851
	0.880	α-terpineol	1199
	0.950	hexyl acetate	1010
Thermal (72 °C for 15 s)	−0.812	(*E*)-2-hexenal	851
	−0.801	α-terpineol	1199
HPP (600 MPa for 3 min)	0.836	2-methylbutyl acetate	877
PEF (15.5 kV/cm)	0.824	hexan-1-ol	869
	0.831	butyl propanoate	906
	0.849	terpinen-4-ol	1185

These volatiles are selected discriminating the process impact immediately after pasteurization. The volatiles are listed in a decreasing order of VID coefficient, where a positive VID coefficient illustrates a higher concentration of a compound after one processing compared to other one and vice versa. The retention index (RI) of compounds is listed. HPP: High-Pressure Processing; PEF: Pulsed Electric Fields; VID: variable identification.

**Table 2:** Volatiles selected by the VID procedure as markers significantly changing as a function of shelf life, in thermal, HPP and PEF pasteurized apple juices.

Processing	VID	Identity	RI
Thermal (72 °C for 15 s)	−0.954	3-methyl-2-buten-1-yl acetate	919
	−0.951	pentyl acetate	911
	−0.948	2-methylpropyl butanoate	943
HPP (600 MPa for 3 min)	−0.971	butyl propanoate	906
	−0.970	pentyl acetate	911
	−0.938	ethyl 2-methylbutanoate	896
	−0.920	hexyl acetate	1010
	−0.841	(*E*)-2-hexenal	851
PEF (15.5 kV/cm)	−0.939	butyl propanoate	906
	−0.878	ethyl 2-methylbutanoate	896
	−0.858	hexyl acetate	1010
	−0.831	hexanal	797

These discriminant markers are listed in increasing order of VID coefficient. Positive VID coefficients signify an increase in concentration during storage while negative coefficients denote a decrease. Their retention index (RI) is also listed.
